# Supplementary material for: Fermentation conditions outweigh phylogeny in shaping the metabolome of novel Micromonospora strains: an integrated genomics-metabolomics analysis
Source: Appl Environ Microbiol. 2026 Jan 26;92(2):e02235-25. doi: 10.1128/aem.02235-25 (PMC12915310; doi:10.1128/aem.02235-25)
Supplement: Supplemental material — Figures S1 to S8; Tables S1 to S6. [file aem.02235-25-s0001.docx]

**Supplementary figures**


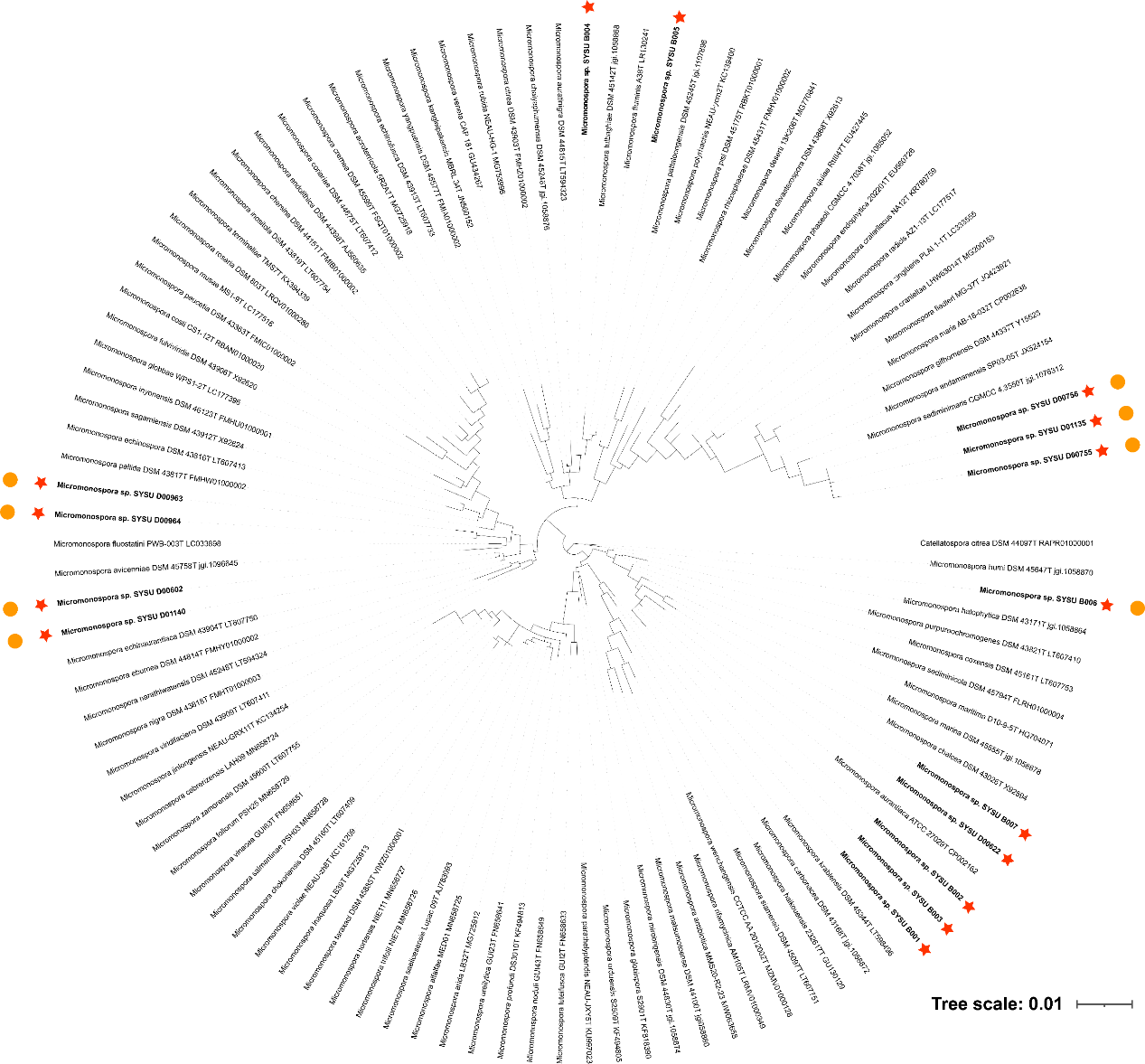


Fig. S1 Maximum-likelihood phylogenetic tree based on 16S rRNA gene sequences showing the relationships of *Micromonospora* isolates and their relatives. *Catellatospora citrea* DSM 44097^T^ was used as the outgroup. Bar, 0.01 substitutions per nucleotide position. The red star symbols at the outer edges of the isolate names represent strains isolated in this study, and the orange circle symbols in the outermost ring represent novel *Micromonospora* isolates.


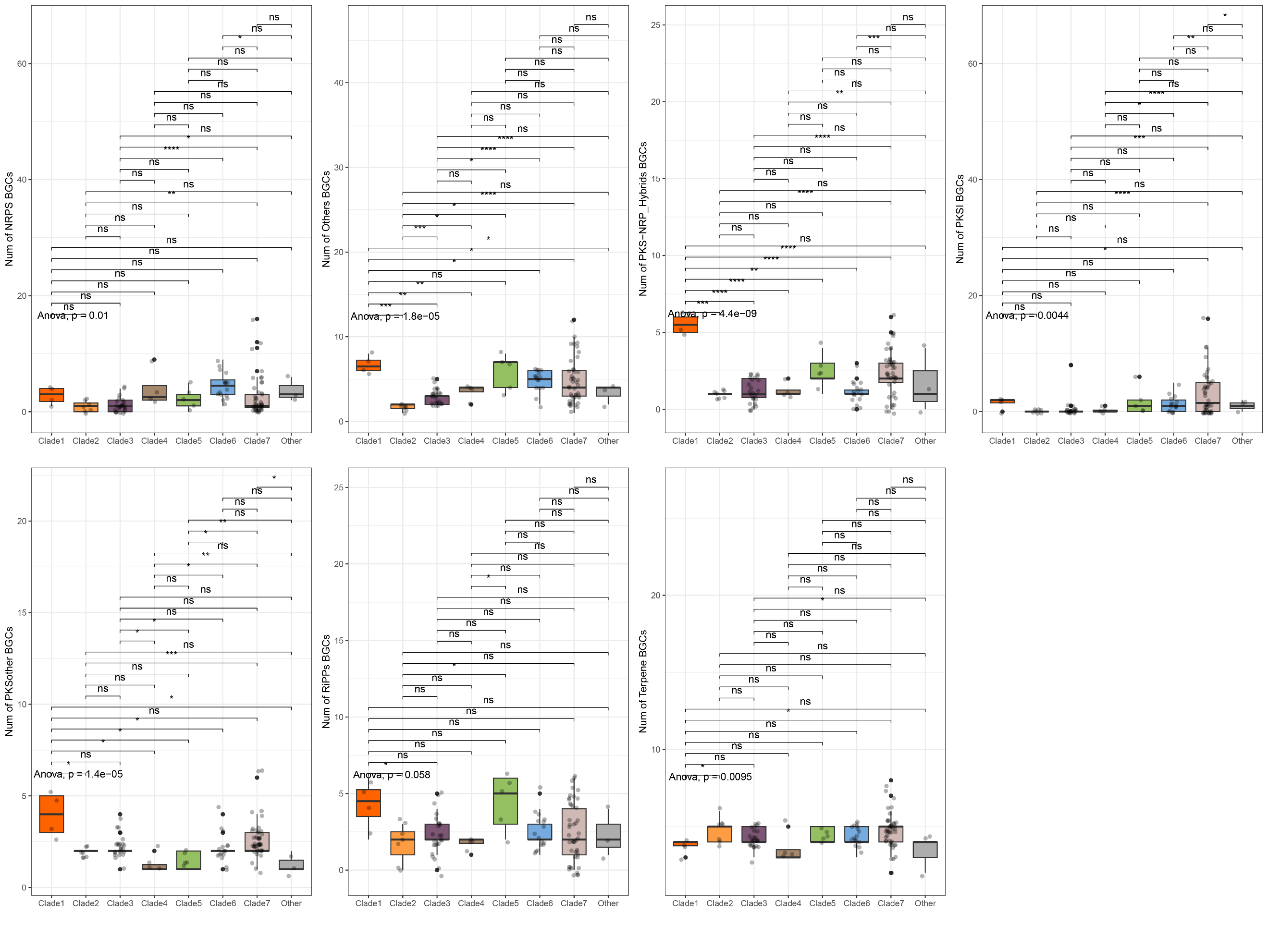


Fig. S2 Scatter plot and box plot of BGC counts in genus *Micromonospora* based on phylogenetic relationships grouping. Significance levels are as follows: *p* > 0.05 = n.s., *p* < 0.5 = *, *p* < 0.01 = **, *p* <0.001 = ***, *p* < 0.0001 = ****.


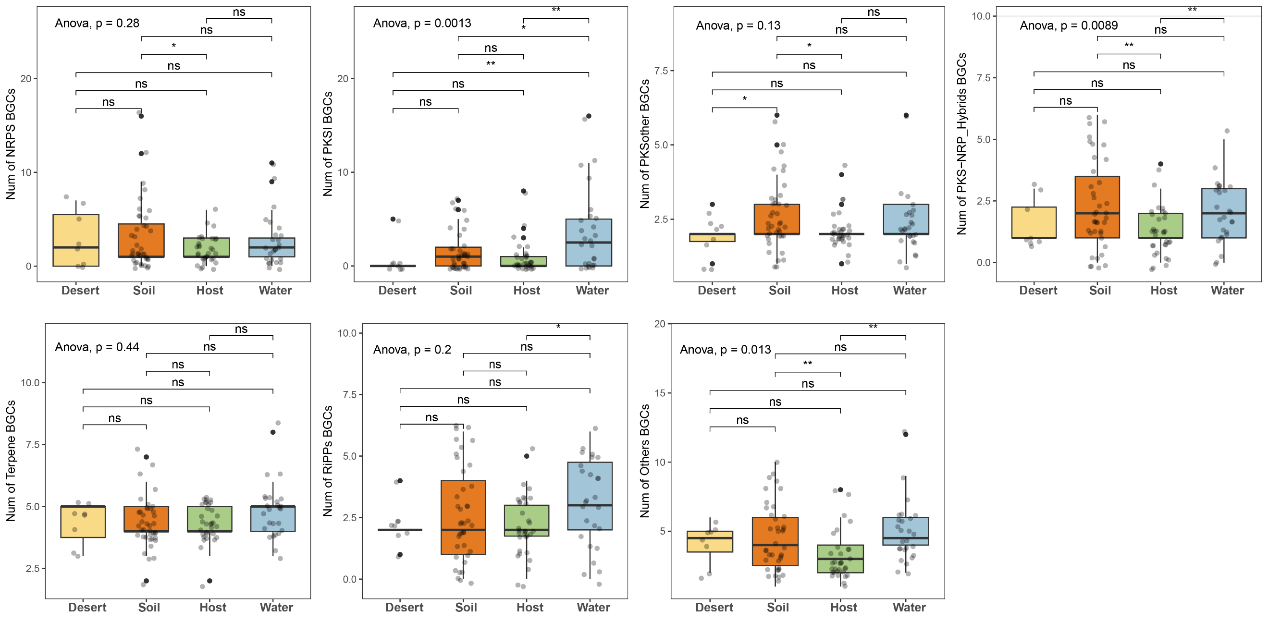


Fig. S3 Scatter plot and box plot of BGC counts in genus *Micromonospora* based on different isolation sources. Significance levels are as follows: *p* > 0.05 = n.s., *p* < 0.5 = *, *p* < 0.01 = **, *p* <0.001 = ***, *p* < 0.0001 = ****.


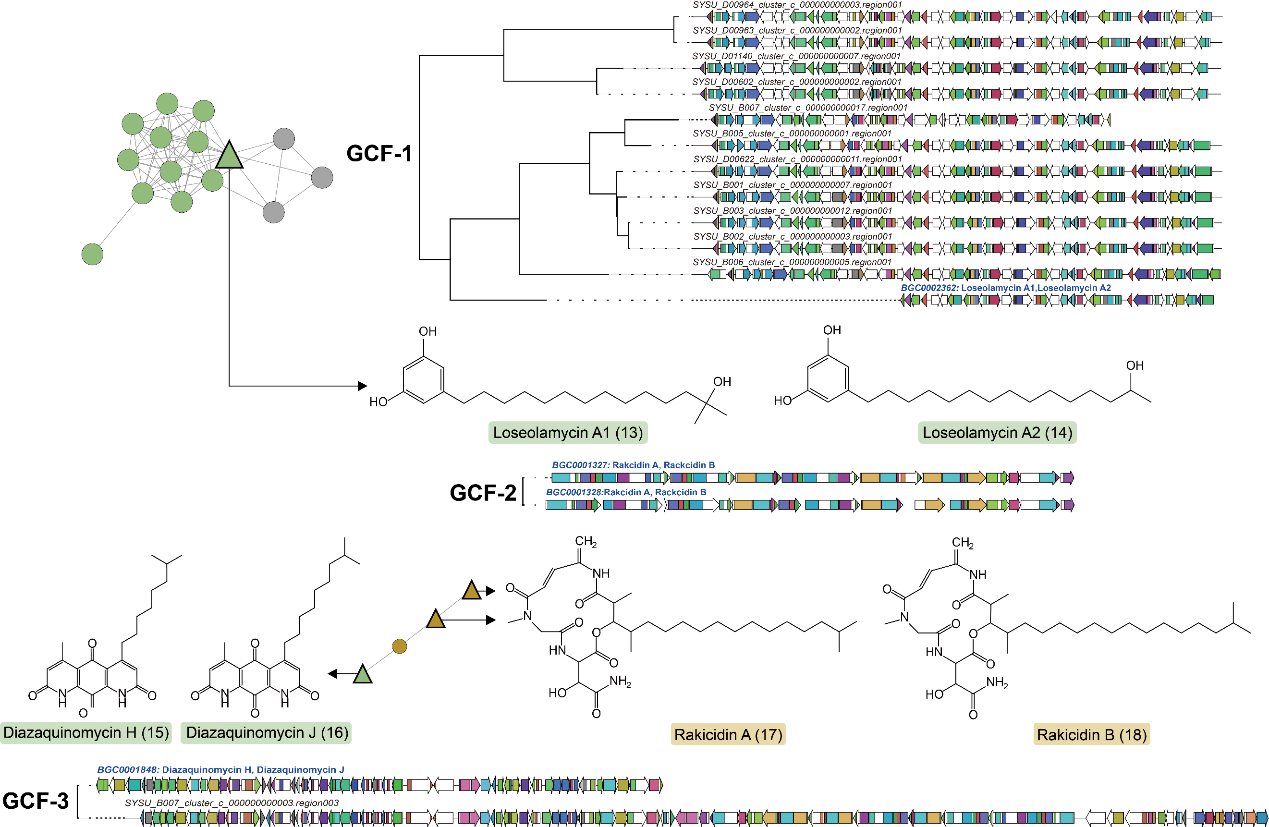


Fig. S4 The GCFs resulted from clustering the BGCs of isolates with reference BGCs from the MIBiG database, along with the natural products encoded by the reference BGCs.


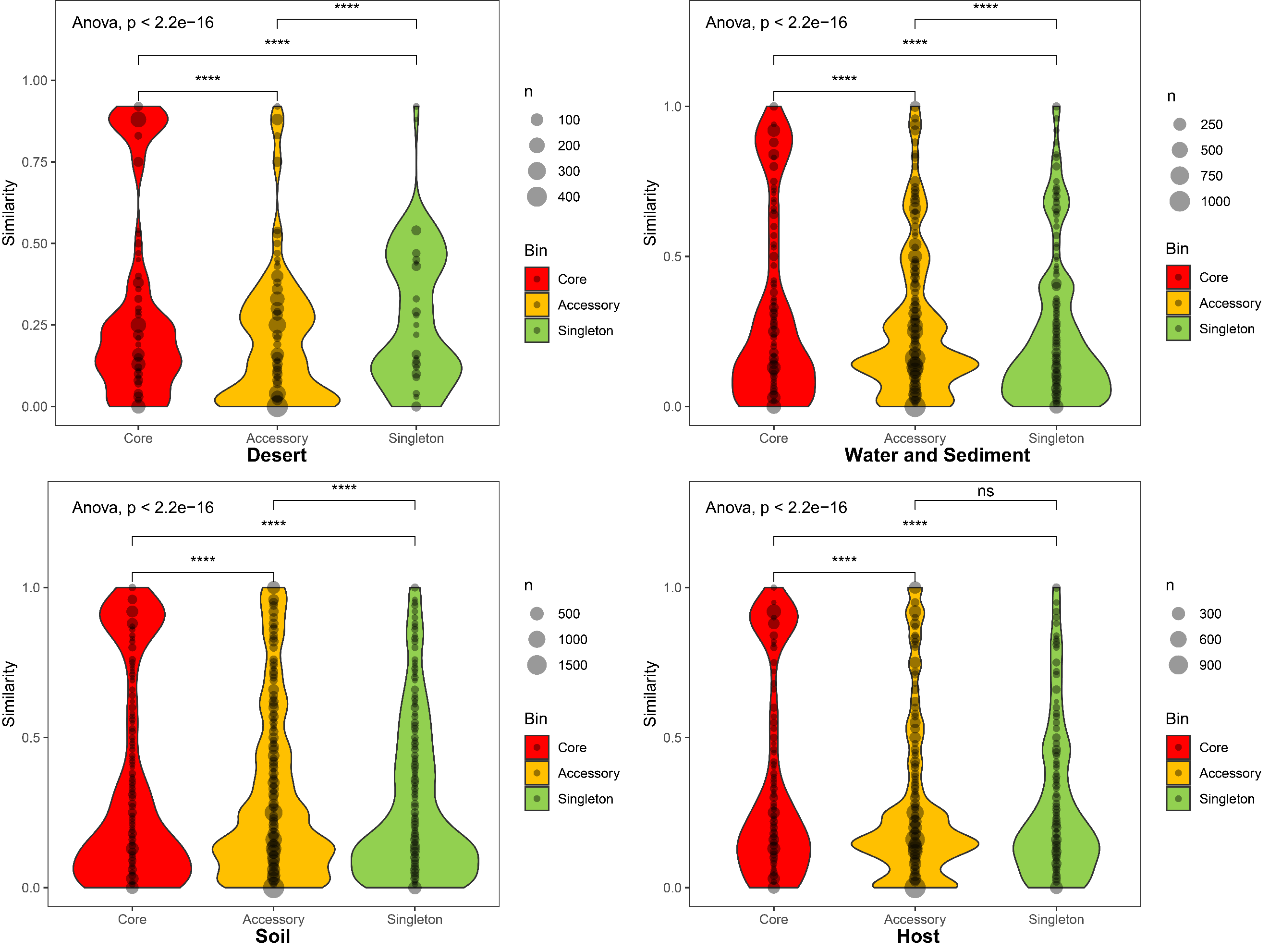


Fig. S5 Violin and bubble plots displaying the similarity of core genes, accessory genes, and singleton genes containing BGs in the pan-genome datasets of different isolation to those encoding known natural products in the database. Significance levels are as follows: *p* > 0.05 = n.s., *p* < 0.5 = *, *p* < 0.01 = **, *p* <0.001 = ***, *p* < 0.0001 = ****.


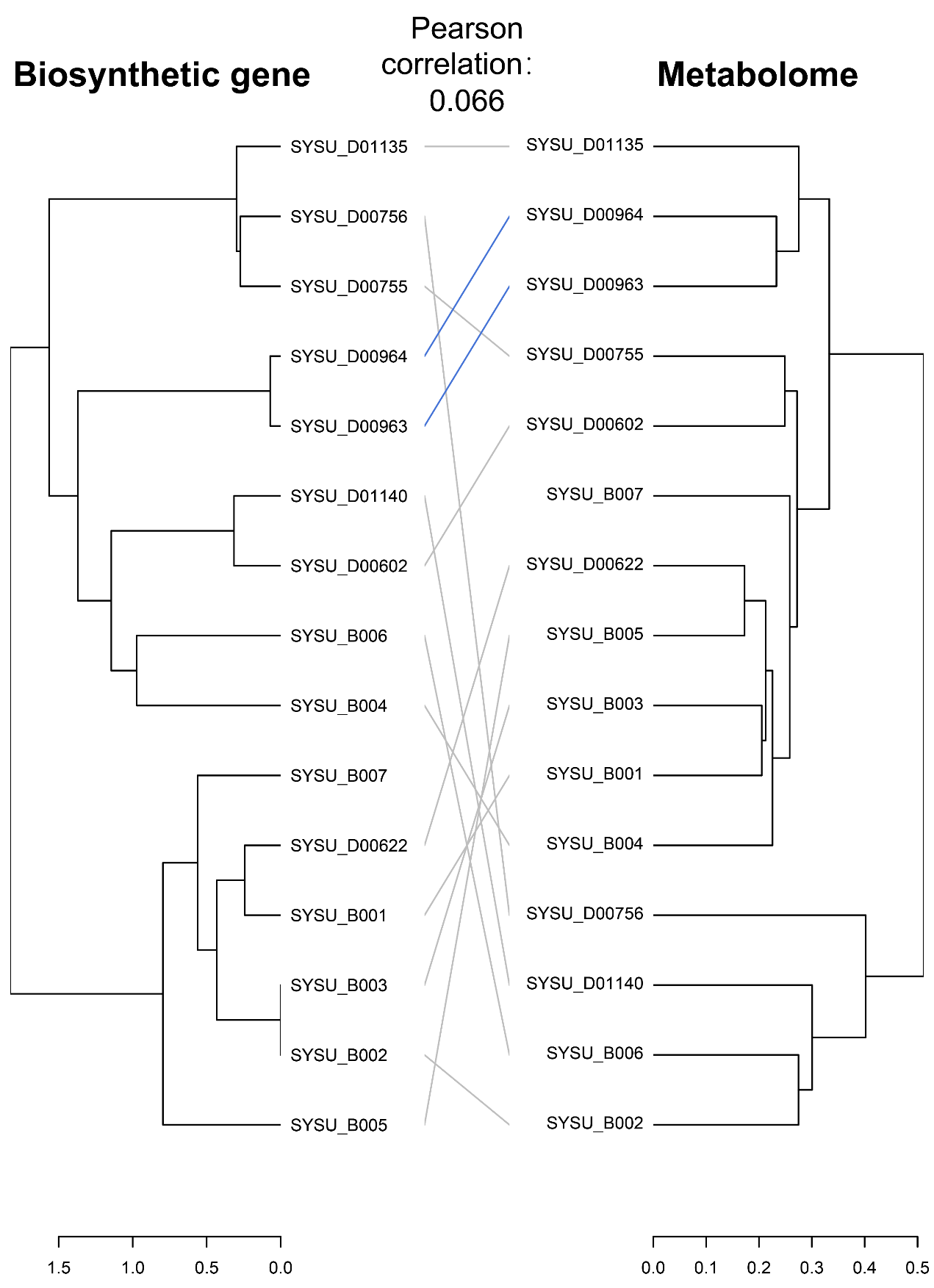


Fig. S6 Comparison of hierarchical clustering trees of the biosynthetic genes and metabolomic data of *Micromonospora* isolates based on Jaccard and Bray-Curtis dissimilarities, respectively, Solid and colored lines connect matching subtrees in the two trees.


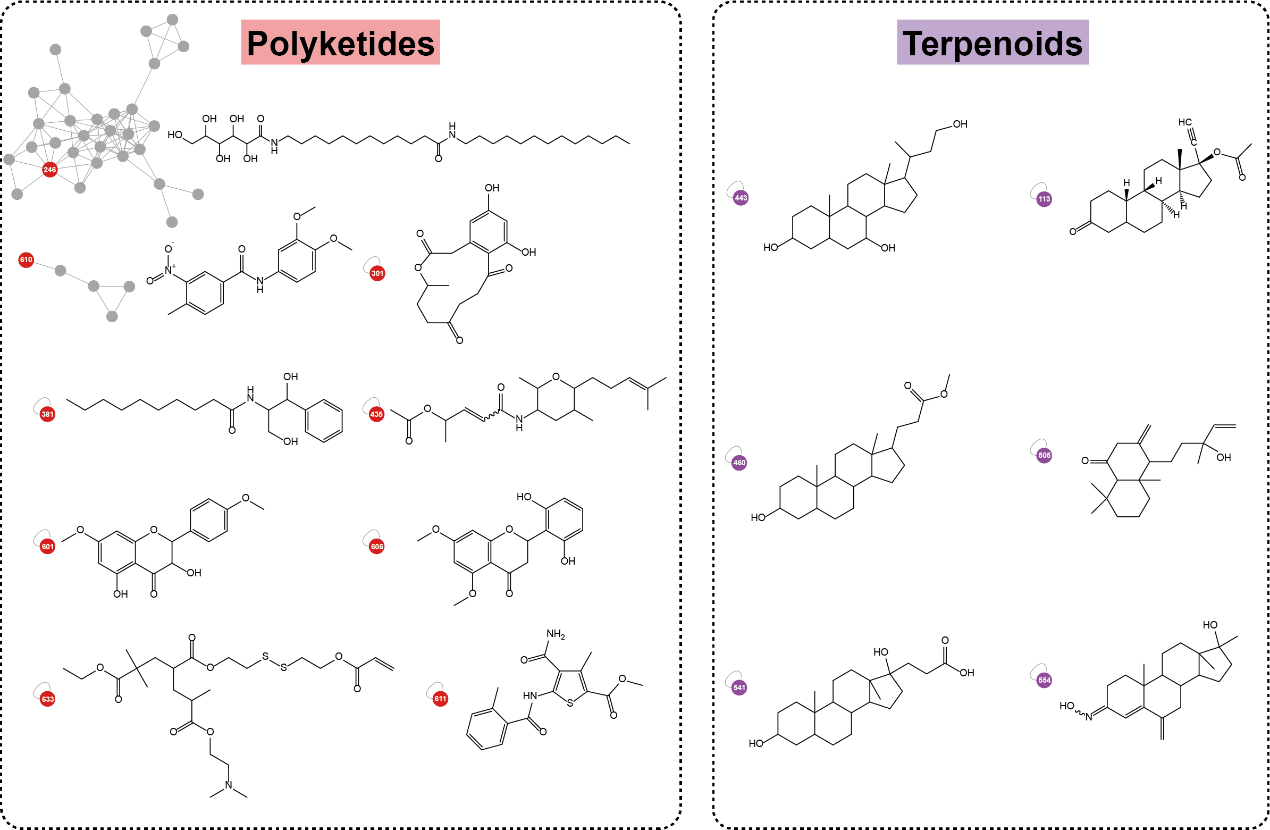


Fig. S7 The features annotated as Polyketides and Terpenoids pathways in the metabolome were processed to obtain molecular structural fingerprints through fragmentation tree computation of MS/MS spectra and were subsequently matched with molecular structures in PubChem.


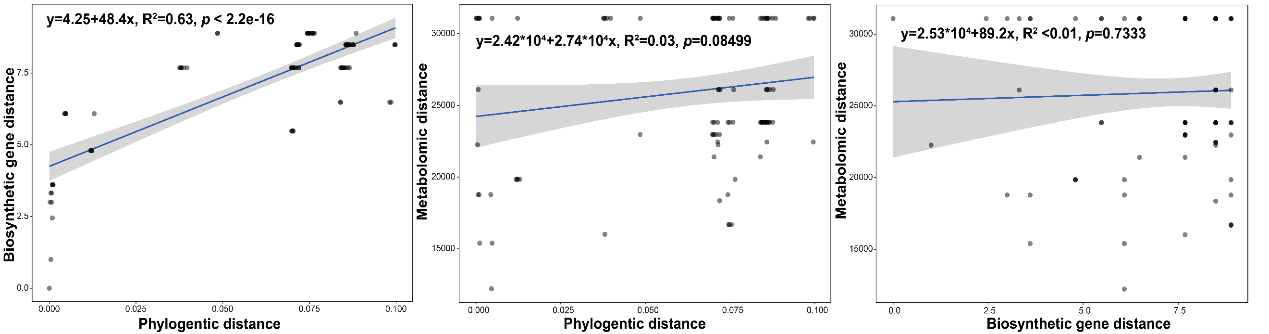


Fig. S8 Correlation between phylogenetic distance and biosynthesis gene distance, between phylogenetic distance and metabolomic distance, and between biosynthesis gene distance and metabolomic distance. Linear model (LM) was used for the correlation analysis and adjustments were made for R2 calculation.

**Supplementary tables**

Table S1 Comparison results of 16S rRNA gene squences of 15 *Micrimonospora* strains.

| Strain | Top-hit strain | Similarity |
| --- | --- | --- |
| SYSU B001 | *Micromonospora aurantiaca* JCM 10878^T^ | 99.65% |
| SYSU B002 | *Micromonospora aurantiaca* JCM 10878^T^ | 99.93% |
| SYSU B003 | *Micromonospora aurantiaca* JCM 10878^T^ | 100.00% |
| SYSU B004 | *Micromonospora fluminis* LMG 30467^T^ | 99.65% |
| SYSU B005 | *Micromonospora fluminis* LMG 30467^T^ | 99.93% |
| SYSU B006 | *Micromonospora humi* JCM 15292^T^ | 99.51% |
| SYSU B007 | *Micromonospora chalcea* CGMCC 4.1050^T^ | 99.72% |
| SYSU D00602 | *Micromonospora echinaurantiaca* JCM 3257^T^ | 99.72% |
| SYSU D00622 | *Micromonospora aurantiaca* JCM 10878^T^ | 99.58% |
| SYSU D00755 | *Micromonospora sediminimaris* CGMCC 4.3550 ^T^ | 99.16% |
| SYSU D00756 | *Micromonospora sediminimaris* CGMCC 4.3550 ^T^ | 99.30% |
| SYSU D00963 | *Micromonospora fluostatini* JCM 30529^T^ | 98.89% |
| SYSU D00964 | *Micromonospora fluostatini* JCM 30529^T^ | 99.16% |
| SYSU D01135 | *Micromonospora sediminimaris* CGMCC 4.3550 ^T^ | 99.30% |
| SYSU D01140 | *Micromonospora echinaurantiaca* JCM 3257^T^ | 99.79% |

Table S2 The ANI values of 15 *Micromonospora* strains.

| Strain | Top-hit strain | ANI |
| --- | --- | --- |
| SYSU B001 | *Micromonospora aurantiaca* ATCC 27029^T^ | 98.81% |
| SYSU B002 | *Micromonospora aurantiaca* ATCC 27029^T^ | 98.71% |
| SYSU B003 | *Micromonospora aurantiaca* ATCC 27029^T^ | 98.70% |
| SYSU B004 | *Micromonospora echinofusca* DSM 43913 | 97.30% |
| SYSU B005 | *Micromonospora aurantiaca* ATCC 27029^T^ | 95.82% |
| SYSU B006 | *Micromonospora humi* DSM 45647^T^ | 91.39% |
| SYSU B007 | *Micromonospora chalcea* DSM 43026^T^ | 98.68% |
| SYSU D00602 | *Micromonospora echinaurantiaca* DSM 43904^T^ | 94.35% |
| SYSU D00622 | *Micromonospora aurantiaca* ATCC 27029^T^ | 98.70% |
| SYSU D00755 | *Micromonospora sediminimaris* CGMCC 4.3550^T^ | 90.14% |
| SYSU D00756 | *Micromonospora sediminimaris* CGMCC 4.3550 ^T^ | 90.16% |
| SYSU D00963 | *Micromonospora globbae* WPS1-2^T^ | 87.99% |
| SYSU D00964 | *Micromonospora globbae* WPS1-2^T^ | 88.10% |
| SYSU D01135 | *Micromonospora sediminimaris* CGMCC 4.3550 ^T^ | 90.11% |
| SYSU D01140 | *Micromonospora echinaurantiaca* DSM 43904^T^ | 94.31% |

Table S3 The dDDH value of novel *Micromonospora* strains.

| Strain | Top-hit strain | dDDH |
| --- | --- | --- |
| SYSU B006 | *Micromonospora humi* DSM 45647^T^ | 42.50% |
| SYSU D00602 | *Micromonospora echinaurantiaca* DSM 43904^T^ | 53.70% |
| SYSU D00755 | *Micromonospora sediminimaris* CGMCC 4.3550^T^ | 38.30% |
| SYSU D00756 | *Micromonospora sediminimaris* CGMCC 4.3550 ^T^ | 38.50% |
| SYSU D00963 | *Micromonospora globbae* WPS1-2^T^ | 32.60% |
| SYSU D00964 | *Micromonospora globbae* WPS1-2^T^ | 32.70% |
| SYSU D01135 | *Micromonospora sediminimaris* CGMCC 4.3550 ^T^ | 38.30% |
| SYSU D01140 | *Micromonospora echinaurantiaca* DSM 43904^T^ | 53.80% |

Table S4 Reference sequences used in this study.

| **Species** | **Type Strain** | **16S rRNA accession number** | **GenBank accession number** |
| --- | --- | --- | --- |
| *Micromonospora acroterricola* | 5R2A7 | MG725918 | GCA_003172955.1 |
| *Micromonospora alfalfae* | MED01 | MN658725 | GCA_022230925.1 |
| *Micromonospora andamanensis* | NBRC 109075 | JX524154 | GCA_016863495.1 |
| *Micromonospora antibiotica* | MMS20-R2-23 | MW063658 | GCA_017599305.1 |
| *Micromonospora arida* | LB32 | MG725912 | GCA_003857035.1 |
| *Micromonospora aurantiaca* | ATCC 27029 | CP002162 | GCA_000145235.1 |
| *Micromonospora auratinigra* | DSM 44815 | LT594323 | GCA_900089595.1 |
| *Micromonospora avicenniae* | DSM 45758 | jgi.1096645 | GCA_900156065.1 |
| *Micromonospora cabrerizensis* | LAH09 | MN658724 | GCA_022230955.1 |
| *Micromonospora carbonacea* | aurantiaca | jgi.1058872 | GCA_013389765.1 |
| *Micromonospora chaiyaphumensis* | DSM 45246 | jgi.1058876 | GCA_900091435.1 |
| *Micromonospora chalcea* | 1K05785M01 | X92594 | GCA_028768405.1 |
| *Micromonospora chersina* | DSM 44151 | FMIB01000002 | GCA_900091475.1 |
| *Micromonospora chokoriensis* | DSM 45160 | LT607409 | GCA_900091505.1 |
| *Micromonospora citrea* | DSM 43903 | FMHZ01000002 | GCA_900090315.1 |
| *Micromonospora coriariae* | DSM 44875 | LT607412 | GCA_900091455.1 |
| *Micromonospora costi* | CS1-12 | RBAN01000020 | GCA_003626655.1 |
| *Micromonospora coxensis* | DSM 45161 | LT607753 | GCA_900090295.1 |
| *Micromonospora craniellae* | LHW63014 | MG200153 | GCA_014764405.1 |
| *Micromonospora craterilacus* | NA12 | KR780759 | GCA_003236315.1 |
| *Micromonospora cremea* | DSM 45599 | FSQT01000002 | GCA_900143515.1 |
| *Micromonospora deserti* | 13K206 | MG770841 | GCA_003236335.1 |
| *Micromonospora eburnea* | DSM 44814 | FMHY01000002 | GCA_900090225.1 |
| *Micromonospora echinaurantiaca* | DSM 43904 | LT607750 | GCA_900090235.1 |
| *Micromonospora echinofusca* | DSM 43913 | LT607733 | GCA_900091445.1 |
| *Micromonospora echinospora* | DSM 43816 | LT607413 | GCA_900091495.1 |
| *Micromonospora endolithica* | DSM 44398 | AJ560635 | GCA_007994185.1 |
| *Micromonospora endophytica* | JCM 18317 | EU560726 | GCA_003581605.1 |
| *Micromonospora fiedleri* | MG-37 | JQ423921 | GCA_016774385.1 |
| *Micromonospora fluminis* | A38 | LR130241 | GCA_902825365.1 |
| *Micromonospora fluostatini* | JCM30529 | LC033898 | GCA_004348605.2 |
| *Micromonospora foliorum* | PSH25 | MN658729 | GCA_022229015.1 |
| *Micromonospora fulviviridis* | JCM 3259 | X92620 | GCA_014648395.1 |
| *Micromonospora gifhornensis* | NBRC 16317 | Y15523 | GCA_016863515.1 |
| *Micromonospora globbae* | WPS1-2 | LC177396 | GCA_003610785.1 |
| *Micromonospora globispora* | S2904 | KF818390 | GCA_003176735.1 |
| *Micromonospora haikouensis* | JXNU-1 | GU130129 | GCA_000876225.1 |
| *Micromonospora halophytica* | DSM 43171 | jgi.1058864 | GCA_900090245.1 |
| *Micromonospora hortensis* | NIE111 | MN658727 | GCA_022230935.1 |
| *Micromonospora humi* | DSM 45647 | jgi.1058870 | GCA_900090105.1 |
| *Micromonospora inaquosa* | LB39 | MG725913 | GCA_003857055.1 |
| *Micromonospora inositola* | DSM 43819 | LT607754 | GCA_900090285.1 |
| *Micromonospora inyonensis* | DSM 46123 | FMHU01000001 | GCA_900091415.1 |
| *Micromonospora jinlongensis* | DSM 45876 | KC134254 | GCA_013410645.1 |
| *Micromonospora kangleipakensis* | DSM 45612 | JN560152 | GCA_004217615.1 |
| *Micromonospora krabiensis* | DSM 45344 | LT598496 | GCA_900091425.1 |
| *Micromonospora luteifusca* | DSM 100204 | FN658633 | GCA_016907275.1 |
| *Micromonospora marina* | DSM 45555 | jgi.1058878 | GCA_900091565.1 |
| *Micromonospora maris* | AB-18-032 | CP002638 | GCA_000204155.1 |
| *Micromonospora maritima* | DSM 45782 | HQ704071 | GCA_902825405.1 |
| *Micromonospora matsumotoense* | DSM 44100 | jgi058880 | GCA_900091525.1 |
| *Micromonospora mirobrigensis* | DSM 44830 | jgi.1058874 | GCA_900091555.1 |
| *Micromonospora musae* | NGC1-4 | LC177516 | GCA_003626545.1 |
| *Micromonospora narathiwatensis* | DSM 45248 | LT594324 | GCA_900089605.1 |
| *Micromonospora nigra* | DSM 43818 | FMHT01000003 | GCA_900091585.1 |
| *Micromonospora noduli* | MED15 | FN658649 | GCA_003264475.1 |
| *Micromonospora olivasterospora* | DSM 43868 | X92613 | GCA_007830265.1 |
| *Micromonospora orduensis* | S2509 | KF494805 | GCA_006228125.1 |
| *Micromonospora pallida* | DSM 43817 | FMHW01000002 | GCA_900090325.1 |
| *Micromonospora parathelypteridis* | CGMCC 4.7347 | KU997023 | GCA_014646315.1 |
| *Micromonospora pattaloongensis* | DSM 45245 | jgi.1107896 | GCA_900107255.1 |
| *Micromonospora peucetia* | DSM 43363 | FMIC01000002 | GCA_900091625.1 |
| *Micromonospora phaseoli* | CGMCC 4.7038 | jgi.1085052 | GCA_900109115.1 |
| *Micromonospora pisi* | DSM 45175 | RBKT01000001 | GCA_003633685.1 |
| *Micromonospora polyrhachis* | DSM 45886 | KC139400 | GCA_014203835.1 |
| *Micromonospora profundi* | DSM 45981 | KF494813 | GCA_011927785.1 |
| *Micromonospora purpureochromogenes* | DSM 43821 | LT607410 | GCA_900091515.1 |
| *Micromonospora qiuiae* | NBRC 106684 | EU427445 | GCA_016863555.1 |
| *Micromonospora radicis* | AZ1-13 | LC177517 | GCA_003583405.1 |
| *Micromonospora rhizosphaerae* | DSM 45431 | FMHV01000002 | GCA_900091465.1 |
| *Micromonospora rifamycinica* | DSM 44983 | LRMV01000349 | GCA_900090265.1 |
| *Micromonospora rosaria* | DSM 803 | LRQV01000286 | GCA_001567585.1 |
| *Micromonospora rubida* | NEAU-HG-1 | MG753996 | GCA_009908295.1 |
| *Micromonospora saelicesensis* | DSM 44871 | AJ783993 | GCA_900091575.1 |
| *Micromonospora sagamiensis* | DSM 43912 | X92624 | GCA_007829995.1 |
| *Micromonospora salmantinae* | PSH03 | MN658728 | GCA_022230905.1 |
| *Micromonospora sediminicola* | DSM 45794 | FLRH01000004 | GCA_900089585.1 |
| *Micromonospora sediminimaris* | NBRC 107745 | jgi.1076312 | GCA_016863575.1 |
| *Micromonospora siamensis* | DSM 45097 | LT607751 | GCA_900090305.1 |
| *Micromonospora taraxaci* | DSM 45885 | VIWZ01000001 | GCA_007830095.1 |
| *Micromonospora terminaliae* | DSM 101760 | KX394339 | GCA_009671205.1 |
| *Micromonospora trifolii* | NIE79 | MN658726 | GCA_022229005.1 |
| *Micromonospora tulbaghiae* | CNY-010 | jgi.1058868 | GCA_003612775.1 |
| *Micromonospora ureilytica* | DSM 101692 | FN658641 | GCA_015751765.1 |
| *Micromonospora veneta* | CAP181 | GU434267 | GCA_016598485.1 |
| *Micromonospora vinacea* | DSM 101695 | FN658651 | GCA_015751785.1 |
| *Micromonospora violae* | DSM 45888 | KC161209 | GCA_004217135.1 |
| *Micromonospora viridifaciens* | DSM 43909 | LT607411 | GCA_900091545.1 |
| *Micromonospora wenchangensis* | CCTCC AA 2012002 | MZMV01000128 | GCA_002210435.1 |
| *Micromonospora yangpuensis* | DSM 45577 | FMIA01000002 | GCA_900091615.1 |
| *Micromonospora zamorensis* | DSM 45600 | LT607755 | GCA_900090275.1 |
| *Micromonospora zingiberis* | PLAI 1-1 | LC333555 | GCA_004331455.1 |
| *Catellatospora citrea* | DSM 44097 | RAPR01000001 | GCA_003610235.1 |

Table S5 Genetic information of isolated strains

| **Species** | **Strain** | **16S rRNA accession number** | **GenBank accession number** | **BioSample accession number** |
| --- | --- | --- | --- | --- |
| *Micromonospora sp.* | SYSU B001 | PP106170 | JAXCFY000000000 | SAMN38324447 |
| *Micromonospora sp.* | SYSU B002 | PP106171 | JAXCFX000000000 | SAMN38324448 |
| *Micromonospora sp.* | SYSU B003 | PP106172 | JAXCFW000000000 | SAMN38324449 |
| *Micromonospora sp.* | SYSU B004 | PP106173 | JAXCFV000000000 | SAMN38324450 |
| *Micromonospora sp.* | SYSU B005 | PP106174 | JAXCFU000000000 | SAMN38324451 |
| *Micromonospora sp.* | SYSU B006 | PP106175 | JAXCFT000000000 | SAMN38324452 |
| *Micromonospora sp.* | SYSU B007 | PP106176 | JAXCFS000000000 | SAMN38324453 |
| *Micromonospora sp.* | SYSU D00602 | PP106163 | JAXCFQ000000000 | SAMN38324455 |
| *Micromonospora sp.* | SYSU D00622 | PP106162 | JAXCFR000000000 | SAMN38324454 |
| *Micromonospora sp.* | SYSU D00755 | PP106167 | JAXCFM000000000 | SAMN38324459 |
| *Micromonospora sp.* | SYSU D00756 | PP106168 | JAXCFL000000000 | SAMN38324460 |
| *Micromonospora sp.* | SYSU D00963 | PP106165 | JAXCFO000000000 | SAMN38324457 |
| *Micromonospora sp.* | SYSU D00964 | PP106166 | JAXCFN000000000 | SAMN38324458 |
| *Micromonospora sp.* | SYSU D01135 | PP106169 | JAXCFK000000000 | SAMN38324461 |
| *Micromonospora sp.* | SYSU D01140 | PP106164 | JAXCFP000000000 | SAMN38324456 |

Table S6 Information of known functional molecules

| **known functional molecules** | **compound number** | **features number** | **source** | **function** |
| --- | --- | --- | --- | --- |
| Dexoyfructosazine | 1 | 17, 25 | All isolates | Flavors in tobacco industry, treatment and prevention of diabetes (type II), resistance of cancers, treatment of immunological and inflammatory disease, reagents for DNA strand cleavage(1) |
| N, N-Diacetylcystine | 2 | 107 | SYSU D01135, SYSU D01140, SYSU D00602, SYSU D00622, SYSU D00755, SYSU D00756, SYSU B001, SYSU B002, SYSU B004, SYSU B005, SYSU B006, SYSU B007 | Treatment of liver injury(2) |
| Enalaprilat | 3 | 110 | SYSU D01135, SYSU D00602, SYSU D00756, SYSU B001, SYSU B002, SYSU B004, SYSU B005 | An intravenously administered angiotensin-converting enzyme inhibitor(3) |
| Norethisterone acetate | 4 | 113 | SYSU D00602, SYSU D00756, SYSU D00963, SYSU B005 | A progestin medication which is used in birth control pills, menopausal hormone therapy, and for the treatment of gynecological disorders(4) |
| Noopept | 5 | 334 | SYSU D01135, SYSU D00756 | Nootropics, therapeutic effects on Alzheimer's disease(5) |
| N-Palmitoylglycine | 6 | 452 | SYSU D01135, SYSU B002 | An effective regulator of pain and inflammation(6) |
| Myristamidopropyl betaine | 7 | 489 | SYSU B001 | A mild surfactant used in washing products(7) |
| Sphinganine | 8 | 493 | All isolates | An intermediate in the production of ceramide(8) |
| N-Oleoylethanolamine | 9 | 524 | All isolates | An endogenous PPAR-alpha agonist produced by adipose tissue that regulates lipolysis by activating PPAR-alpha. It plays an anti-atherosclerotic role by reducing oxidation, inflammation and hyperlipidemia(9) |
| Erucamide | 10 | 578 | All isolates | Mainly used as anti-slip additive, anti-blocking agent, as well as paper coating composition and waterproof(10) |
| 3,5-Dihydroxy-4',7-dimethoxyflavone | 11 | 601 | All isolates | Antioxidant, antityrosinase and anti-inflammatory compounds(11) |
| Icosa Sphingosine | 12 | 615 | SYSU D00755, SYSU B007 | A highly bioactive compound that is involved in a variety of cellular processes, including cellular interactions, cell proliferation, differentiation, and apoptosis(12) |

**Reference**

1. Wu S, Fan H, Zhang Q, Cheng Y, Wang Q, Yang G, Han B. 2011. Conversions of Cellobiose and Inulin to Deoxyfructosazine in Aqueous Solutions. Clean-soil Air Water 39:572-576.

2. Wang F, Liu S, Zhuang R, Bao J, Shen Y, Xi J, Sun J, Fang H. 2018. N,N'-diacetylcystine ameliorates inflammation in experimental non-alcoholic steatohepatitis by regulating nuclear transcription factor kappa B activation. Int J Clin Exp Pathol 11:5351-5358.

3. Strauss R, Gavras I, Vlahakos D, Gavras H. 1986. Enalaprilat in Hypertensive Emergencies. The Journal of Clinical Pharmacology 26:39-43.

4. Huvinen E, Holopainen E, Heikinheimo O. 2021. Norethisterone and its acetate – what’s so special about them? BMJ Sexual &amp; Reproductive Health 47:102-109.

5. Ostrovskaya RU, Gruden MA, Bobkova NA, Sewell RDE, Gudasheva TA, Samokhin AN, Seredinin SB, Noppe W, Sherstnev VV, Morozova-Roche LA. 2007. The nootropic and neuroprotective proline-containing dipeptide noopept restores spatial memory and increases immunoreactivity to amyloid in an Alzheimer's disease model. Journal of Psychopharmacology 21:611-619.

6. Rimmerman N, Bradshaw HB, Hughes HV, Chen JS-C, Hu SS-J, McHugh D, Vefring E, Jahnsen JA, Thompson EL, Masuda K, Cravatt BF, Burstein S, Vasko MR, Prieto AL, O'Dell DK, Walker JM. 2008. <em>N</em>-Palmitoyl Glycine, a Novel Endogenous Lipid That Acts As a Modulator of Calcium Influx and Nitric Oxide Production in Sensory Neurons. Molecular Pharmacology 74:213-224.

7. Burnett CL, Bergfeld WF, Belsito DV, Hill RA, Klaassen CD, Liebler D, Marks JG, Shank RC, Slaga TJ, Snyder PW, Andersen FA. 2012. Final Report of the Cosmetic Ingredient Review Expert Panel on the Safety Assessment of Cocamidopropyl betaine (CAPB). International Journal of Toxicology 31:77S-111S.

8. Lambeth JD, Burnham DN, Tyagi SR. 1988. Sphinganine effects on chemoattractant-induced diacylglycerol generation, calcium fluxes, superoxide production, and on cell viability in the human neutrophil. Delivery of sphinganine with bovine serum albumin minimizes cytotoxicity without affecting inhibition of the respiratory burst. J Biol Chem 263:3818-22.

9. Grijalvo S, Bedia C, Triola G, Casas J, Llebaria A, Teixidó J, Rabal O, Levade T, Delgado A, Fabriàs G. 2006. Design, synthesis and activity as acid ceramidase inhibitors of 2-oxooctanoyl and N-oleoylethanolamine analogues. Chemistry and Physics of Lipids 144:69-84.

10. Molnar NM. 1974. Erucamide. Journal of the American Oil Chemists' Society 51:84-87.

11. Gupta MK, Senthilkumar S, Chiranjivi AK, Banik K, Girisa S, Kunnumakkara AB, Dubey VK, Rangan L. 2021. Antioxidant, anti-tyrosinase and anti-inflammatory activities of 3,5-dihydroxy-4′,7-dimethoxyflavone isolated from the leaves of Alpinia nigra. Phytomedicine Plus 1:100097.

12. Brogden G, Husein DM, Steinberg P, Naim HY. 2019. Isolation and Quantification of Sphingosine and Sphinganine from Rat Serum Revealed Gender Differences. Biomolecules 9:459.
